# Transabdominal approach laparoscopic ureteral reimplantation at the top of the bladder for the treatment of primary obstructive megaureter

**DOI:** 10.3389/fped.2025.1552433

**Published:** 2025-04-07

**Authors:** Xianhui Shang, Zhen Luo, Yingbo Li, Guangxu Zhou, Yuchen Mao, Hongyang Tan, Kaiyi Mao, Peng Zhao, Cao Wang, Zhu Jin, Yuanmei Liu

**Affiliations:** ^1^ Department of Pediatric Surgery, Affiliated Hospital of Zunyi Medical University, Zunyi, China; ^2^ Department of Pediatric Surgery, Guizhou Children’s Hospital, Zunyi, China

**Keywords:** congenital primary obstructive megaureter (POM), transabdominal laparoscopic ureteral reimplantation (TALUR), politano procedure, posterior wall-bladder dome, hydronephrosis, vesicoureteral reflux (VUR), minimally invasive surgery, ureteral dilation

## Abstract

**Background:**

Congenital primary obstructive megaureter (POM) is characterized by distal ureteral obstruction, leading to ureteral dilation, hydronephrosis, and potential renal impairment. Surgical intervention is necessary for severe hydronephrosis (SFU grade III–IV) or progressive renal decline. Open ureteral reimplantation is the standard treatment but is associated with significant surgical trauma and prolonged recovery. This study evaluates the safety and efficacy of transabdominal laparoscopic ureteral reimplantation (TALUR) at the posterior wall-bladder dome and compares its outcomes with the Politano procedure.

**Methods:**

This retrospective, single-center study included pediatric POM patients who underwent ureteral reimplantation at the Affiliated Hospital of Zunyi Medical University from October 2019 to December 2023. Patients were assigned to the TALUR group (*n* = 21) or the Politano group (*n* = 20). Preoperative imaging, including renal ultrasound, magnetic resonance urography (MRU), and voiding cystourethrography (VCUG), confirmed the diagnosis. Primary endpoints included postoperative distal ureteral diameter, renal pelvic diameter, surgical success rate, perioperative complications, hospital stay, and vesicoureteral reflux (VUR) incidence. Follow-up assessments included ultrasound, MRU, and VCUG.

**Results:**

All procedures were successfully completed without conversion to open surgery. The TALUR group had a significantly shorter operative time (76.5 ± 12.6 min) compared to the Politano group (95.7 ± 14.8 min, *P* < 0.05). Postoperatively, distal ureteral diameter decreased from 14.6 ± 3.7 mm–4.8 ± 2.1 mm (*P* < 0.05), and renal pelvic dilation improved from 24.7 ± 5.3 mm–12.3 ± 2.6 mm (*P* < 0.05). The TALUR group had a shorter hospital stay (4.5 ± 0.5 vs. 6.1 ± 0.7 days, *P* < 0.05). Follow-up MRU showed improved ureteral patency and resolution of hydronephrosis. VCUG at six months showed mild VUR in two TALUR patients (9.5%) and one Politano patient (5.0%), all resolving within one year.

**Conclusion:**

TALUR is a safe and effective minimally invasive technique for pediatric POM. Compared to the Politano procedure, TALUR offers shorter operative time, faster recovery, and comparable efficacy. Further large-scale studies are required to confirm its long-term effectiveness.

## Introduction

Congenital primary obstructive megaureter (POM) is a common cause of obstructive uropathy in pediatric patients, characterized by functional obstruction at the distal ureter, leading to progressive ureteral dilation, hydronephrosis, and potential renal function impairment (1). Without timely intervention, POM may result in irreversible kidney damage, making surgical treatment necessary for patients with severe hydronephrosis [Society for Fetal Urology (SFU) grade III–IV] or progressive renal function decline (2).

Traditionally, open ureteral reimplantation has been the standard surgical treatment for POM. Although effective, it is associated with significant surgical trauma, prolonged hospitalization, and a high risk of postoperative complications (3). With the advancement of minimally invasive techniques, laparoscopic ureteral reimplantation (TALUR) has gained increasing attention due to its advantages, including reduced surgical trauma, faster recovery, and improved cosmetic outcomes (4, 5). However, performing laparoscopic ureteral reimplantation at the posterior bladder wall presents certain technical challenges, primarily due to limited surgical exposure and the complexity of ureteral anastomosis, which restricts its widespread application (6).

The Politano-Leadbetter procedure is a widely used intravesical ureteral reimplantation technique that has demonstrated excellent outcomes in preventing vesicoureteral reflux (VUR). However, this approach requires extensive bladder wall dissection and results in a longer operative time, making it less favorable for minimally invasive surgery (7). To overcome these limitations, transabdominal laparoscopic ureteral reimplantation (TALUR) has been introduced as an alternative approach, providing improved surgical visualization, easier ureteral mobilization, and more controlled anastomosis (8).

Despite the advantages of TALUR, its application at the posterior bladder wall remains challenging due to restricted working space and the technical difficulty of creating an adequate submucosal tunnel to achieve an anti-reflux effect. To address these challenges, we modified the TALUR technique by repositioning the ureteral reimplantation site to the posterior wall-bladder dome. This modification enhances surgical exposure, simplifies anastomosis, and optimizes the anti-reflux mechanism (9).

This study aims to evaluate the safety and efficacy of TALUR at the posterior wall-bladder dome in pediatric patients with POM and to compare its clinical outcomes with the Politano procedure. By analyzing postoperative improvements in ureteral dilation, hydronephrosis resolution, surgical success rates, and perioperative complications, we aim to determine whether this modified laparoscopic approach is a viable alternative to traditional intravesical ureteral reimplantation.

## Methods

### Study design and participants

This retrospective, single-center comparative study was conducted at the Affiliated Hospital of Zunyi Medical University between October 2019 and December 2023. The study aimed to evaluate the safety and efficacy of transabdominal laparoscopic ureteral reimplantation (TALUR) at the posterior wall-bladder dome in pediatric patients with primary obstructive megaureter (POM) and to compare its clinical outcomes with the Politano procedure.

All patients underwent comprehensive preoperative evaluations, including renal ultrasound, magnetic resonance urography (MRU), voiding cystourethrography (VCUG), and differential renal function assessment to confirm the diagnosis of POM and determine the severity of hydronephrosis.

A total of 41 pediatric patients were included and categorized into two groups based on the surgical approach: the TALUR group (*n* = 21) and the Politano procedure group (*n* = 20). Inclusion Criteria: (1) SFU Grade III–IV hydronephrosis. (2) Progressive renal function decline confirmed by diuretic renography. (3) No prior surgical treatment for POM. Exclusion Criteria: (1) Bilateral ureteral stenosis. (2) Secondary causes of ureteral obstruction (e.g., trauma, malignancy). (3) Severe comorbidities precluding laparoscopic surgery. The study was approved by the institutional review board, and informed consent was obtained from all guardians before enrollment.

## Surgical technique

### Preoperative preparation

All patients underwent standard preoperative preparation, including bowel preparation with a preoperative enema to reduce postoperative abdominal distension. Antibiotic prophylaxis was administered with cephalosporins 30 min before surgery to minimize the risk of postoperative infection. Preoperative imaging evaluation, including renal ultrasound, magnetic resonance urography (MRU), and voiding cystourethrography (VCUG), was performed to identify the location of ureteral stenosis and assess the severity of hydronephrosis.

### Surgeon and surgical approach

All procedures were performed by a single experienced laparoscopic pediatric urologist proficient in transabdominal laparoscopic ureteral reimplantation (TALUR). The patient was placed in the Trendelenburg position (15° head-down tilt) with a slight contralateral tilt to optimize the surgical field. A Foley catheter was inserted preoperatively for continuous bladder drainage. Pneumoperitoneum was established through a 5 mm umbilical port for CO₂ insufflation, maintaining an intra-abdominal pressure of 10–12 mmHg. Trocar placement included a 5 mm umbilical port for the camera and two additional 5 mm working ports at the midclavicular line at the level of the umbilicus. No 3 mm ports were used, as they provide less stability for precise suturing.

### Ureteral dissection and lesion confirmation

The exact location of ureteral stenosis and the degree of distal ureteral dilation were confirmed through intraoperative laparoscopic exploration in combination with preoperative imaging. Ureteral dissection commenced at the level of the iliac vessels and proceeded distally toward the bladder wall. The stenotic distal ureteral segment, approximately 3–4 cm in length, was completely excised to ensure unobstructed urinary passage following reimplantation. The proximal ureteral stump was obliquely incised to widen the anastomotic opening and reduce the risk of stricture.

### Bladder preparation and submucosal tunnel construction

The posterior wall-bladder dome was selected as the reimplantation site to optimize surgical exposure and reduce anastomotic complexity. A submucosal tunnel was created using an ultrasonic scalpel to make a longitudinal incision in the bladder seromuscular layer and dissect the submucosal layer. The tunnel was constructed with a length-to-diameter ratio of 5:1 to ensure an effective anti-reflux mechanism. The bladder mucosa was preserved to facilitate smooth ureteral passage and secure anastomosis.

### Ureteral reimplantation and suturing

The distal ureter was inserted obliquely into the submucosal tunnel to minimize vesicoureteral reflux (VUR). Mucosa-to-mucosa anastomosis was performed using 5-0 absorbable sutures (PDS or Vicryl) to prevent anastomotic stenosis. The bladder seromuscular layer was closed with 4-0 barbed sutures, covering the ureter to reinforce the anti-reflux mechanism.

### Ureteral tailoring and double-J stent placement

In cases of ureteral wall thickening or rigidity, intraoperative tailoring was performed to improve postoperative ureteral patency. The pre-trim ureteral distal diameter measured 14.7 ± 3.9 mm, and post-trim diameter measured 4.7 ± 2.2 mm, ensuring optimal luminal patency and preventing stenosis. The double-J stent was introduced via a contralateral abdominal wall puncture using a 5 mm hollow needle to guide the stent into the peritoneal cavity, followed by laparoscopic-assisted insertion into the reimplanted ureter. 3 Fr stents were avoided due to concerns over limited drainage capacity. The stent was retrogradely inserted through the ureteral orifice, with the proximal end positioned in the renal pelvis and the distal end in the bladder, confirmed by cystoscopy. Untrimmed ureters were generally not stented to minimize foreign body-related complications.

### Postoperative assessment and drainage

Anastomotic integrity was verified intraoperatively by filling the bladder with saline to detect leaks, ensuring a watertight closure. A pelvic drainage tube was placed to monitor urinary leakage, and if no significant leakage was noted within 3 days postoperatively, the drain was removed. The Foley catheter was maintained for 5 days postoperatively and then removed. The double-J stent remained in place for 4 weeks and was removed at follow-up to prevent prolonged stent-related complications.

### Postoperative follow-up and evaluation

Ultrasound evaluations were conducted at 1, 3, and 6 months postoperatively to monitor ureteral dilation improvement and hydronephrosis resolution. VCUG was selectively performed at 6 months to assess the presence of occult VUR. Surgical success was defined by the absence of ureteral stricture, urinary fistula, or urinary tract infection, along with significant improvement in ureteral dilation and hydronephrosis and no new-onset VUR on postoperative VCUG.

## Conclusion

**Figure 1:**
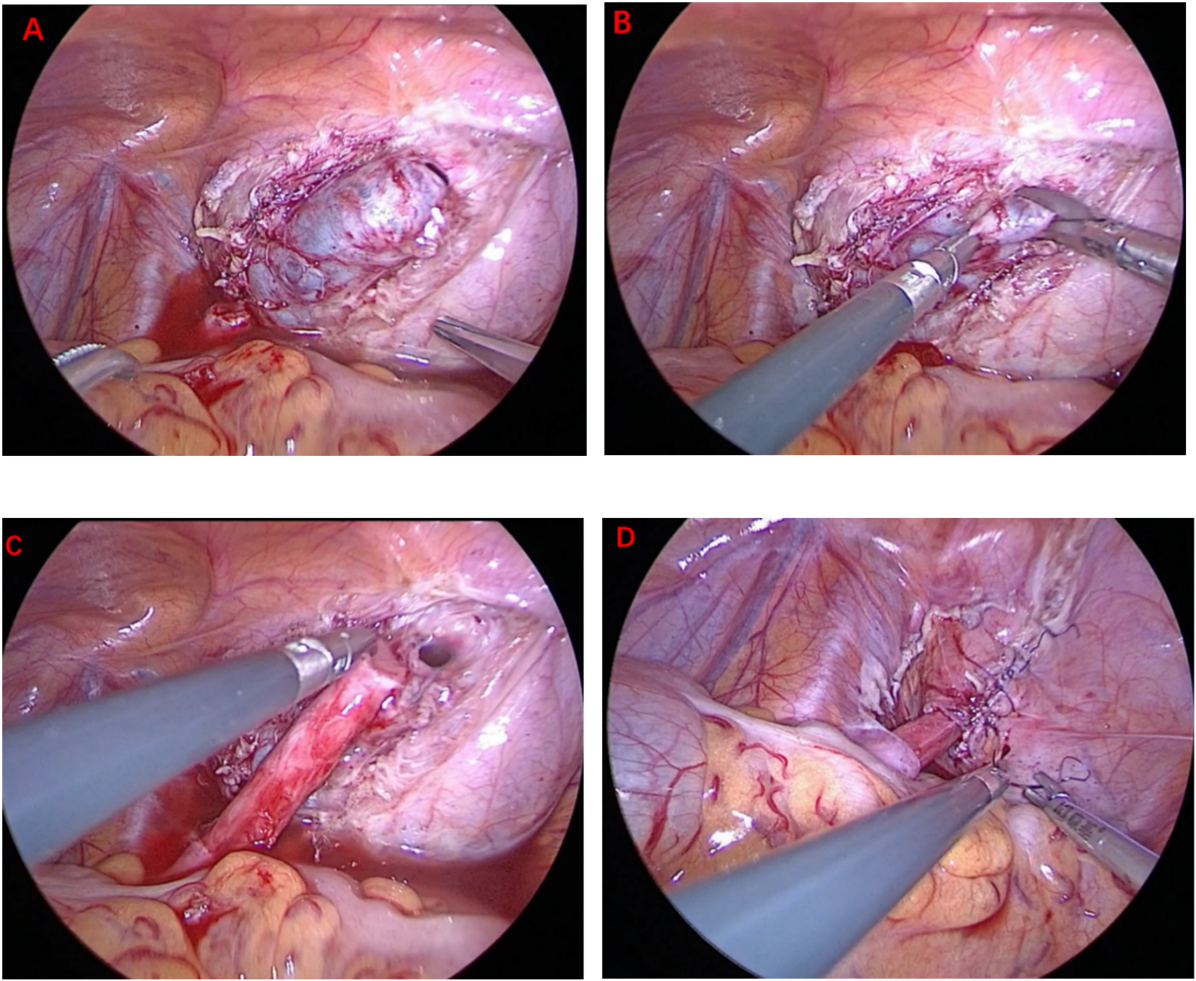
Surgical procedure of TALUR for treating left-side POM: **(A)** creation of a submucosal tunnel **(B)** opening of the bladder mucosa at the dome. **(C)** anastomosis of the ureter to the bladder mucosa **(D)** closure of the bladder.

TALUR at the posterior wall-bladder dome provides a minimally invasive alternative to traditional Politano ureteral reimplantation, integrating a submucosal tunnel (5:1 ratio) for effective anti-reflux protection. Compared to the Politano procedure, TALUR is associated with faster recovery, reduced surgical trauma, and fewer complications, making it a viable surgical option for pediatric POM patients. This technique also allows for simultaneous bilateral ureteral reimplantation, enhancing procedural efficiency and optimizing clinical outcomes. Further multicenter, prospective studies are necessary to validate its long-term efficacy (Figure 1).

## Results

### Patient characteristics

This study included 41 pediatric patients diagnosed with primary obstructive megaureter (POM) who underwent transabdominal laparoscopic ureteral reimplantation (TALUR) at the posterior wall-bladder dome or the Politano procedure. The TALUR group comprised 21 patients, while the Politano group included 20 patients. The mean age of all patients was 31.1 ± 18.1 months (range: 12–75 months), with 25 males (61.0%) and 16 females (39.0%). Preoperative imaging assessments, including renal ultrasound, magnetic resonance urography (MRU), diuretic renography, and voiding cystourethrography (VCUG), confirmed significant distal ureteral obstruction in all cases. The baseline patient characteristics are summarized in Table 1.

**Table 1:** Patient demographics and preoperative characteristics.

Variable	TALUR group (*n* = 21)	Politano group (*n* = 20)
Mean Age (months)	30.8 ± 17.9 (12–72)	31.4 ± 18.5 (13–75)
Male/Female Ratio	13/8 (61.9%/38.1%)	12/8 (60.0%/40.0%)
SFU Grade III Hydronephrosis	9 (42.9%)	9 (45.0%)
SFU Grade IV Hydronephrosis	12 (57.1%)	11 (55.0%)
Mean Preoperative Distal Ureter Diameter (mm)	14.6 ± 3.7	14.8 ± 4.1
Mean Preoperative Renal Pelvic Diameter (mm)	24.7 ± 5.3	25.1 ± 5.0
Patients with Ureteral Wall Thickening/Rigidity	3 (14.3%)	2 (10.0%)

### Intraoperative and postoperative outcomes

All 41 surgeries were successfully completed without conversion to open surgery. The mean operative time was 76.5 ± 12.6 min (range: 58–110 min) in the TALUR group and 95.7 ± 14.8 min (range: 73–122 min) in the Politano group (*P* < 0.05), demonstrating a significantly shorter duration for the TALUR technique.

Three patients in the TALUR group had thickened and rigid distal ureters, necessitating externalization through a port, trimming, and reintroduction for reimplantation. The mean postoperative hospital stay was 4.5 ± 0.5 days (range: 4–5 days) in the TALUR group, significantly shorter than 6.1 ± 0.7 days (range: 5–7 days) in the Politano group (*P* < 0.05). Three patients in the TALUR group who required ureteral tailoring had double-J stents placed intraoperatively, and all stents were successfully removed within four weeks.

During the follow-up period (mean: 15 months; range: 4–25 months), ultrasonographic and MRU evaluations demonstrated significant reductions in distal ureteral dilation and hydronephrosis in both groups. In the TALUR group, the distal ureteral diameter decreased from 14.6 ± 3.7 mm–4.8 ± 2.1 mm (*P* < 0.05), and renal pelvic diameter reduced from 24.7 ± 5.3 mm–12.3 ± 2.6 mm (*P* < 0.05). Similar improvements were observed in the Politano group, with no statistically significant differences between the two groups (*P* > 0.05). Table 2 presents the intraoperative and postoperative outcomes.

**Table 2:** Intraoperative and postoperative outcomes.

Variable	TALUR Group (*n* = 21)	Politano Group (*n* = 20)	*P*-value
Mean Operative Time (min)	76.5 ± 12.6	95.7 ± 14.8	<0.05
Mean Hospital Stay (days)	4.5 ± 0.5	6.1 ± 0.7	<0.05
Postoperative Double-J Stent Placement	3 (14.3%)	2 (10.0%)	0.62
Mean Reduction in Distal Ureter Diameter (mm)	9.8 ± 3.9	9.6 ± 4.2	0.75
Mean Reduction in Renal Pelvic Diameter (mm)	12.4 ± 5.1	12.7 ± 4.9	0.81

### Postoperative MRU and VUR follow-up

MRU follow-up was conducted at 1, 3, and 6 months postoperatively, demonstrating significant improvement in ureteral patency and marked resolution of hydronephrosis in all patients. Additionally, preoperative MRU assessments revealed that in some patients, distal ureteral dilation resulted in bladder compression and morphological alterations, including bladder wall thinning, reduced bladder capacity, and asymmetry in bladder shape.

Postoperative MRU evaluations indicated that bladder compression was significantly alleviated following ureteral decompression. In both the TALUR and Politano groups, the bladder regained a more symmetric spherical or elliptical shape, with bladder wall thickness returning to normal and bladder compliance showing improvement. These findings suggest a positive recovery of bladder morphology and function following surgery.

Additionally, selective VCUG was performed at 6 months postoperatively to assess vesicoureteral reflux (VUR). In the TALUR group, 2 patients (9.5%) developed mild VUR postoperatively, but follow-up VCUG at 1 year confirmed complete resolution. Similarly, in the Politano group, 1 patient (5.0%) developed mild VUR, which also resolved at the 1-year follow-up. No cases of severe or persistent VUR were observed in either group.

In summary, TALUR not only effectively improves ureteral dilation and hydronephrosis but also contributes to the restoration of bladder morphology. Compared to the Politano procedure, TALUR exhibits comparable outcomes in postoperative bladder dynamics and anti-reflux function, further supporting its clinical feasibility (Table 3).

**Table 3:** Postoperative MRU and VUR follow-up.

Variable	TALUR group (*n* = 21)	Politano group (*n* = 20)
MRU Improvement in Hydronephrosis	21 (100%)	20 (100%)
Postoperative Mild VUR Cases	2 (9.5%)	1 (5.0%)
Resolution of VUR at 1-Year Follow-up	2/2 (100%)	1/1 (100%)
Bladder Shape Improvement on MRU	18 (85.7%)	17 (85.0%)
Bladder Compliance Improvement	19 (90.5%)	18 (90.0%)

### Complications and perioperative morbidity

No major perioperative complications, such as urinary tract infections (UTIs), anastomotic strictures, voiding dysfunction, ureteral obstruction, or urinary leakage, were observed in either group. In the TALUR group, no febrile UTIs or postoperative infections were reported. In the Politano group, 3 patients (15.0%) developed minor infections at the cystostomy site after catheter removal, which resolved after 15 days of wound care. Additionally, 1 patient (5.0%) in the Politano group experienced mild VUR, which resolved within 1 year (Table 4).

**Table 4:** Complications and morbidity.

Complication	TALUR group (*n* = 21)	Politano group (*n* = 20)
Febrile UTI	0 (0%)	0 (0%)
Non-febrile UTI	0 (0%)	3 (15.0%)
Anastomotic Stricture	0 (0%)	0 (0%)
Voiding Dysfunction	0 (0%)	0 (0%)
Ureteral Obstruction	0 (0%)	0 (0%)
Urinary Leakage	0 (0%)	0 (0%)
Mild VUR	2 (9.5%)	1 (5.0%)
Resolution of VUR (1 Year)	2/2 (100%)	1/1 (100%)

## Summary of key findings

1. 100% success rate with no conversions to open surgery.
2. Shorter operative time and hospital stay in the TALUR group compared to the Politano group (*P* < 0.05).
3. Significant improvements in ureteral dilation and renal pelvic diameter in both groups.
4. No major perioperative complications or persistent VUR in either group.
5. TALUR demonstrated comparable efficacy to the Politano procedure, with faster recovery and minimal morbidity.

These findings suggest that TALUR at the posterior wall-bladder dome is a safe and effective minimally invasive surgical option for pediatric POM, offering advantages over traditional intravesical ureteral reimplantation while maintaining similar surgical efficacy.

### Statistical analysis

Paired t-tests demonstrated significant postoperative improvements in clinical outcomes for both the TALUR and Politano groups. In the TALUR group, the distal ureteral diameter showed a mean reduction of 9.8 ± 3.9 mm, decreasing from 14.6 ± 3.7 mm preoperatively to 4.8 ± 2.1 mm postoperatively (*P* < 0.05). Similarly, the renal pelvic anteroposterior diameter decreased by 12.4 ± 5.1 mm, from 24.7 ± 5.3 mm–12.3 ± 2.6 mm (*P* < 0.05). Comparable improvements were observed in the Politano group, with a mean reduction of 9.6 ± 4.2 mm in distal ureteral diameter (*P* < 0.05) and 12.7 ± 4.9 mm in renal pelvic diameter (*P* < 0.05), with no statistically significant difference between the two groups (*P* > 0.05).

During the follow-up period (mean: 15 months), all patients demonstrated significant ureteral patency improvement and resolution of hydronephrosis, as confirmed by MRU and ultrasonographic assessments. VCUG evaluations at six months postoperatively revealed that two patients (9.5%) in the TALUR group developed mild VUR, while one patient (5.0%) in the Politano group exhibited mild VUR. All cases of VUR resolved spontaneously within one year (Table 5).

**Table 5:** Comparison of preoperative and postoperative measurements.

Variable	TALUR group (*n* = 21)	Politano group (*n* = 20)	*P*-value
Preoperative Distal Ureter Diameter (mm)	14.6 ± 3.7	14.8 ± 4.1	0.79
Postoperative Distal Ureter Diameter (mm)	4.8 ± 2.1	5.2 ± 2.3	0.65
Reduction in Distal Ureter Diameter (mm)	9.8 ± 3.9	9.6 ± 4.2	0.75
Preoperative Renal Pelvic Diameter (mm)	24.7 ± 5.3	25.1 ± 5.0	0.68
Postoperative Renal Pelvic Diameter (mm)	12.3 ± 2.6	12.4 ± 3.0	0.84
Reduction in Renal Pelvic Diameter (mm)	12.4 ± 5.1	12.7 ± 4.9	0.81
Postoperative VUR Occurrence	2 (9.5%)	1 (5.0%)	0.63
VUR Resolution at 1-Year Follow-up	2/2 (100%)	1/1 (100%)	-

This study confirms that TALUR performed at the posterior wall-bladder dome is a safe and effective surgical approach for pediatric POM, providing significant reductions in ureteral dilation and hydronephrosis, with outcomes comparable to the Politano procedure. Key findings include:

1. 100% success rate, with no conversions to open surgery in either group.
2. Statistically significant improvements in ureteral diameter and renal pelvic dilation, with no significant difference between the two groups (*P* > 0.05).
3. No major perioperative or short-term complications, and no cases of persistent or severe VUR.
4. TALUR resulted in a significantly shorter operative time (*P* < 0.05) and hospital stay (*P* < 0.05) compared to the Politano procedure, facilitating faster postoperative recovery.

These findings suggest that TALUR represents a viable minimally invasive alternative to traditional intravesical ureteral reimplantation (Politano procedure), offering comparable surgical outcomes while minimizing surgical trauma. Further multicenter, large-sample, and long-term follow-up studies are warranted to fully evaluate its efficacy and potential expansion of indications.

## Discussion

Primary obstructive megaureter (POM) is often detected during prenatal screening, and surgical intervention is typically deferred until the child reaches one year of age. Both international and domestic guidelines recommend performing ureteral reimplantation for POM after the age of one since the small bladder volume in infants under one year may hinder the formation of an adequate submucosal tunnel length-to-ureteral diameter ratio during surgery (10, 11). Ureteral reimplantation is necessary for POM patients with preserved renal function to excise the stenotic ureteral segment and reconstruct a patent urinary outflow tract (12).

Historically, open surgery has been the standard treatment for POM. In 1994, Ehrlich et al. first reported laparoscopic ureteral reimplantation (13). However, the technical challenges of intracorporeal ureteral suturing limited its widespread adoption. With advancements in surgical techniques and instrumentation, laparoscopic approaches have achieved outcomes comparable to open surgery (14, 15). Additionally, laparoscopic surgery has evolved from primarily ablative procedures to reconstructive techniques, including ureteral reimplantation and pyeloplasty (16, 17), offering substantial advantages in upper urinary tract reconstruction (18).

Despite these advancements, ureteral reimplantation remains a technically demanding procedure, particularly at the ureter-to-bladder anastomosis. Traditional laparoscopic and robotic-assisted methods lack the tactile feedback of open surgery, and the limited surgical space further challenges the surgeon's skill (19, 20). The Lich-Gregoir technique, widely used in open surgery, provides excellent visualization and anatomical restoration by implanting the ureter at the posterior lateral bladder wall, with an anastomosis at the lower end of the newly created tunnel (21). However, this technique increases procedural complexity and extends the learning curve. Gander et al. proposed a modification in which the tunnel is created at the ventral aspect of the posterior bladder wall, allowing for a longitudinal incision in the seromuscular layer and utilizing the protruding mucosa for ureteral anastomosis. This modification enhances the operative field and reduces anastomotic difficulty (22, 23).

In this study, we further optimized the procedure by shifting the ureteral reimplantation site to the posterior wall-bladder dome (TALUR), which improves surgical visibility and simplifies anastomosis, particularly in cases requiring ureteral tailoring. Our results demonstrate no perioperative complications, such as urinary tract infections, anastomotic leakage, or voiding dysfunction, supporting the safety profile of TALUR. Previous studies suggest that routine postoperative voiding cystourethrography (VCUG) and renal function assessments are unnecessary in asymptomatic patients, with additional imaging reserved for cases in which ultrasound suggests potential obstruction (24, 25). In our study, MRU and VCUG were selectively performed in addition to routine ultrasound follow-ups. During a mean follow-up of 15.0 ± 5.8 months, ureteral patency and hydronephrosis resolution were consistently observed, further confirming the efficacy of TALUR.

### Innovative aspects and comparison with Lich-Gregoir technique

The primary innovation of TALUR lies in modifying the ureteral implantation site compared to the Lich-Gregoir technique. TALUR places the ureter at the posterior wall-bladder dome, a non-physiological site that may impact long-term bladder dynamics and increase the risk of vesicoureteral reflux (VUR). The Lich-Gregoir technique utilizes a long submucosal tunnel to prevent reflux, whereas long-term data on TALUR's anti-reflux efficacy and its effects on bladder compliance and detrusor function remain limited. Further large-scale studies with long-term follow-up are required to validate its durability and reflux prevention effectiveness.

### Limitations

This study has several limitations. First, the retrospective design may introduce selection bias. Second, the sample size (*n* = 41) is relatively small, which may limit the generalizability of our findings. Additionally, while the follow-up period was sufficient to evaluate short- and mid-term outcomes, it may not fully capture long-term complications or recurrence. Future prospective, multicenter studies with larger sample sizes are needed to further validate the efficacy of TALUR and to establish comprehensive diagnostic and treatment guidelines.

Future research should focus on optimizing surgical techniques to enhance applicability across diverse clinical scenarios. Moreover, comparative studies assessing TALUR against other minimally invasive methods, such as robotic-assisted ureteral reimplantation, are warranted to explore potential refinements in surgical strategies and improve patient outcomes.

## Conclusion

This study demonstrates that TALUR at the posterior wall-bladder dome is a safe and effective minimally invasive technique for pediatric POM. By improving surgical exposure, reducing technical complexity, and increasing anastomotic efficiency, TALUR offers an alternative to open surgery with no perioperative or short-term complications and favorable postoperative outcomes. Continued follow-up into adolescence and adulthood is necessary to evaluate long-term results and potential complications.

## Data Availability

The original contributions presented in the study are included in the article/supplementary material, further inquiries can be directed to the corresponding author.
